# Propranolol can correct prolonged QT intervals in patients with cirrhosis

**DOI:** 10.3389/fphar.2024.1370261

**Published:** 2024-04-26

**Authors:** Huanqin Han, Junlian Chen, Zhirong Deng, Tingting Li, Xiaoying Qi, Wei Deng, Zunge Wu, Chuli Xiao, Weiqiang Zheng, Yujun Du

**Affiliations:** ^1^ Department of Infectious Diseases and Hepatology, Affiliated Hospital of Guangdong Medical University, Zhanjiang, Guangdong, China; ^2^ Cardiovascular Center, Affiliated Hospital of Guangdong Medical University, Zhanjiang, Guangdong, China

**Keywords:** electrocardiography, esophageal and gastric varices, gastrointestinal hemorrhage, cirrhosis, long QT syndrome, propranolol

## Abstract

**Background:**

Prolonged QT intervals are extremely common in patients with cirrhosis and affect their treatment outcomes. Propranolol is often used to prevent gastroesophageal variceal hemorrhage in patients with cirrhosis; however, it is uncertain whether propranolol exerts a corrective effect on QT interval prolongation in patients with cirrhosis.

**Aim:**

The study aimed to investigate the therapeutic effects of propranolol on patients with cirrhosis and prolonged QT intervals.

**Methods:**

A retrospective cohort study approach was adopted. Patients with cirrhosis complicated by moderate-to-severe gastroesophageal varices, who were hospitalized at the Affiliated Hospital of Guangdong Medical University between 1 December 2020 and 31 November 2022, were included in the study. The patients were divided into the propranolol and control groups based on whether they had received propranolol. Upon admission, the patients underwent tests on liver and kidney functions, electrolytes, and coagulation function, as well as abdominal ultrasonography and electrocardiography. In addition to conventional treatment, the patients were followed up after the use or non-use of propranolol for treatment and subsequently underwent reexamination of the aforementioned tests.

**Results:**

The propranolol group (26 patients) had an average baseline corrected QT (QTc) interval of 450.23 ± 37.18 ms, of which 14 patients (53.8%) exhibited QTc interval prolongation. Follow-up was continued for a median duration of 7.00 days after the administration of propranolol and conventional treatment. Electrocardiographic reexamination revealed a decrease in the QTc interval to 431.04 ± 34.64 ms (*p* = 0.014), and the number of patients with QTc interval prolongation decreased to five (19.2%; *p* < 0.001). After treatment with propranolol and multimodal therapy, QTc interval normalization occurred in nine patients with QTc interval prolongation, leading to a normalization rate of 64.3% (9/14). The control group (n = 58) had an average baseline QTc interval of 453.74 ± 30.03 ms, of which 33 patients (56.9%) exhibited QTc interval prolongation. After follow-up for a median duration of 7.50 days, the QTc interval was 451.79 ± 34.56 ms (*p* = 0.482), and the number of patients with QTc interval prolongation decreased to 30 (51.7%; *p* = 0.457). The QTc interval normalization rate of patients in the control group with QTc interval prolongation was merely 10.0% (3/33), which was significantly lower than that in the propranolol group (*p* < 0.001).

**Conclusion:**

In patients with cirrhosis complicated by QT interval prolongation, the short-term use of propranolol aids in correction of a long QT interval and provides positive therapeutic value for cirrhotic cardiomyopathy.

## 1 Introduction

Cirrhotic cardiomyopathy (CCM) refers to the occurrence of chronic cardiac dysfunction in patients with cirrhosis. CCM is a common complication in patients with cirrhosis and is characterized by the development of myocardial contraction dysfunction, diastolic dysfunction, and concomitant electrophysiological abnormalities in the absence of other known heart diseases. However, the clinical manifestations of CCM are insidious, with most patients experiencing a lack of apparent symptoms during the early stage and slow disease progression. This has resulted in low levels of vigilance and a general lack of awareness of the disease among clinicians. Cardiac complications, such as heart failure, may occur in the advanced stage of CCM, thereby affecting the outcomes of patients with cirrhosis (Hachulla et al., 2019; Izzy et al., 2020).

The QT interval on an electrocardiogram (ECG) refers to the duration from the start of the QRS complex to the end of the T wave; it reflects the total time required for ventricular depolarization and repolarization. Given that the QT interval is affected by the heart rate, the corrected QT (QTc) interval is typically used to determine if there is prolongation of the QT interval. The most common electrophysiological abnormality in CCM is prolongation of the QTc interval on an ECG, which occurs in approximately 30%–60% of patients with cirrhosis (Hansen et al., 2007; Kosar et al., 2007; Lee et al., 2022; Papadopoulos and Mimidis, 2023). Prolongation of the QTc interval is known to increase the risk of severe ventricular arrhythmias in patients without cirrhosis. Although fatal arrhythmias are not a common cause of death in patients with cirrhosis, the clinical benefits of improving long QTc intervals in patients with cirrhosis are uncertain. However, the prolongation of the QTc interval is closely related to the severity of cirrhosis and is a predictor of mortality in cirrhosis, especially in patients with gastrointestinal bleeding or those who have undergone transjugular intrahepatic portosystemic shunt placement or liver transplantation (Day et al., 1993; Biselli et al., 2019; Brabrand et al., 2020; Hela et al., 2020).

There is currently no gold standard for the diagnosis of CCM, so a specific and effective medication for its treatment is lacking; however, QTc interval prolongation can be easily diagnosed through ECG examination. QTc prolongation is a supportive criterion of CCM, although such prolongation does not indicate the presence of CCM. A study by Went et al. (2021) reported that nadolol and propranolol, which are non-selective β-blockers, exert therapeutic effects on congenital long QT syndrome. It is widely believed that QTc interval prolongation in patients with cirrhosis is closely associated with CCM, degree of liver dysfunction, electrolyte abnormalities (including hypokalemia and hypocalcemia), portal hypertension, and alcohol toxicity (Bernardi et al., 1998; Hansen et al., 2007; Henriksen et al., 2007). Therefore, the therapeutic effects of propranolol on QT interval prolongation in patients with cirrhosis remain unclear.

Gastroesophageal variceal rupture and hemorrhage are common complications of cirrhosis. The use of non-selective β-blockers or endoscopic variceal ligation is recommended in patients with cirrhosis who are prone to moderate-to-severe gastroesophageal varices and a high risk of hemorrhage for preventing first-time or recurrent hemorrhage (Chinese Society of Hepatology, 2022). Propranolol as a non-selective β-blocker can block the cardiac β_1_ receptors, thereby reducing the cardiac output; this blocks the reflexivity of the β_2_ receptors in the visceral blood vessels, thereby causing vasoconstriction in the visceral arteries and leading to alleviation of portal hypertension in patients with cirrhosis (Zacharias et al., 2018). Propranolol possesses advantages such as low cost, high accessibility, and non-invasiveness, which contribute to its wide usage among patients with gastroesophageal varices, and is still recommended by Chinese guidelines (Chinese Society of Hepatology, 2022); if propranolol enables shortening of the prolonged QT interval, it will serve a dual purpose in these patients. Therefore, the present study aimed to investigate the effects of propranolol on the QTc intervals of patients with cirrhosis complicated by moderate-to-severe gastroesophageal varices. The recommended therapeutic drugs as per Chinese guidelines also include naphthalol, which is widely used in patients with esophageal and gastric varices in cirrhosis.

## 2 Materials and methods

### 2.1 Subjects

Patients with cirrhosis complicated by moderate-to-severe gastroesophageal varices who sought medical attention at the Affiliated Hospital of Guangdong Medical University between 1 December 2020 and 31 November 2022 were included in this study. All patients received treatment for cirrhosis after inclusion. The patients were grouped on the basis of whether they received propranolol; those who underwent treatment with propranolol (10 mg twice daily) were defined as the propranolol group, whereas patients who did not receive propranolol or other non-selective beta-blocker treatment served as the control group.

### 2.2 Inclusion and exclusion criteria

The inclusion criteria were as follows: age ≥18 years and cirrhosis complicated by moderate-to-severe gastroesophageal varices. The exclusion criteria were as follows: bronchial asthma; severe lower-respiratory-tract infection that may be accompanied by dyspnea, sinus bradycardia, second- or third-degree atrioventricular block, cardiogenic shock, severe or acute heart failure, ischemic heart disease, or patients otherwise unsuited for the use of propranolol; concomitant hepatocellular carcinoma; cholangiocarcinoma or ampullary carcinoma; and concomitant portal vein thrombosis.

The diagnostic criteria for cirrhosis with moderate-to-severe gastroesophageal varices included a diagnosis of cirrhosis based on etiology, medical history, clinical presentations, complications, treatment process, laboratory tests, imaging examinations, and histological examinations (Chinese Society of Hepatology, 2019). All enrolled patients underwent gastroscopy within 6 months to confirm the presence of moderate-to-severe gastroesophageal varices (Chinese Society of Hepatology, 2022). QT interval prolongation was defined in this study as a QTc interval exceeding 450 ms in male individuals and 460 ms in female individuals on the ECG. The Bazett formula was included because it is the most commonly used formula in clinical practice, although its adequacy is largely questionable (Zambruni et al., 2007).

### 2.3 Study methods

A retrospective cohort study design was adopted. Upon admission and after posttreatment follow-up, all patients underwent routine examinations, including tests for electrolytes; liver, kidney, and coagulation functions; abdominal color Doppler ultrasonography; and ECG. The patients were also observed for the presence of propranolol-induced symptoms, such as bronchospasms and bradycardia. All enrolled patients received multimodal therapy, which includes treatment of the cause (e.g., alcohol withdrawal, antiviral treatment), endoscopic variceal ligation (if necessary), correction of electrolytic disturbances, and symptomatic treatment according to the guidelines (Chinese Society of Hepatology, 2019) for cirrhosis, excluding surgical treatment. The control group did not receive any β-blockers.

### 2.4 Ethical review

This study was approved by the ethics committee of the Affiliated Hospital of Guangdong Medical University (Approval No. PJKT 2024-040). Informed consent was obtained from all participating subjects in accordance with the guidelines of the Declaration of Helsinki.

### 2.5 Statistical analysis

The data were statistically analyzed using SPSS 25.0. The normally distributed continuous data were expressed as mean ± standard deviation (*x ± s*). Comparisons between the two groups were performed using the *t*-test. Continuous data that did not follow a normal distribution were expressed in terms of the median (interquartile range), and comparisons between the groups were performed using the rank-sum test. The data counts were expressed as percentages, and comparisons between the groups were performed using the χ^2^ test or Fisher’s exact test. Differences were considered to be statistically significant for *p* < 0.05.

## 3 Results

### 3.1 Subject inclusion process

**FIGURE 1:**
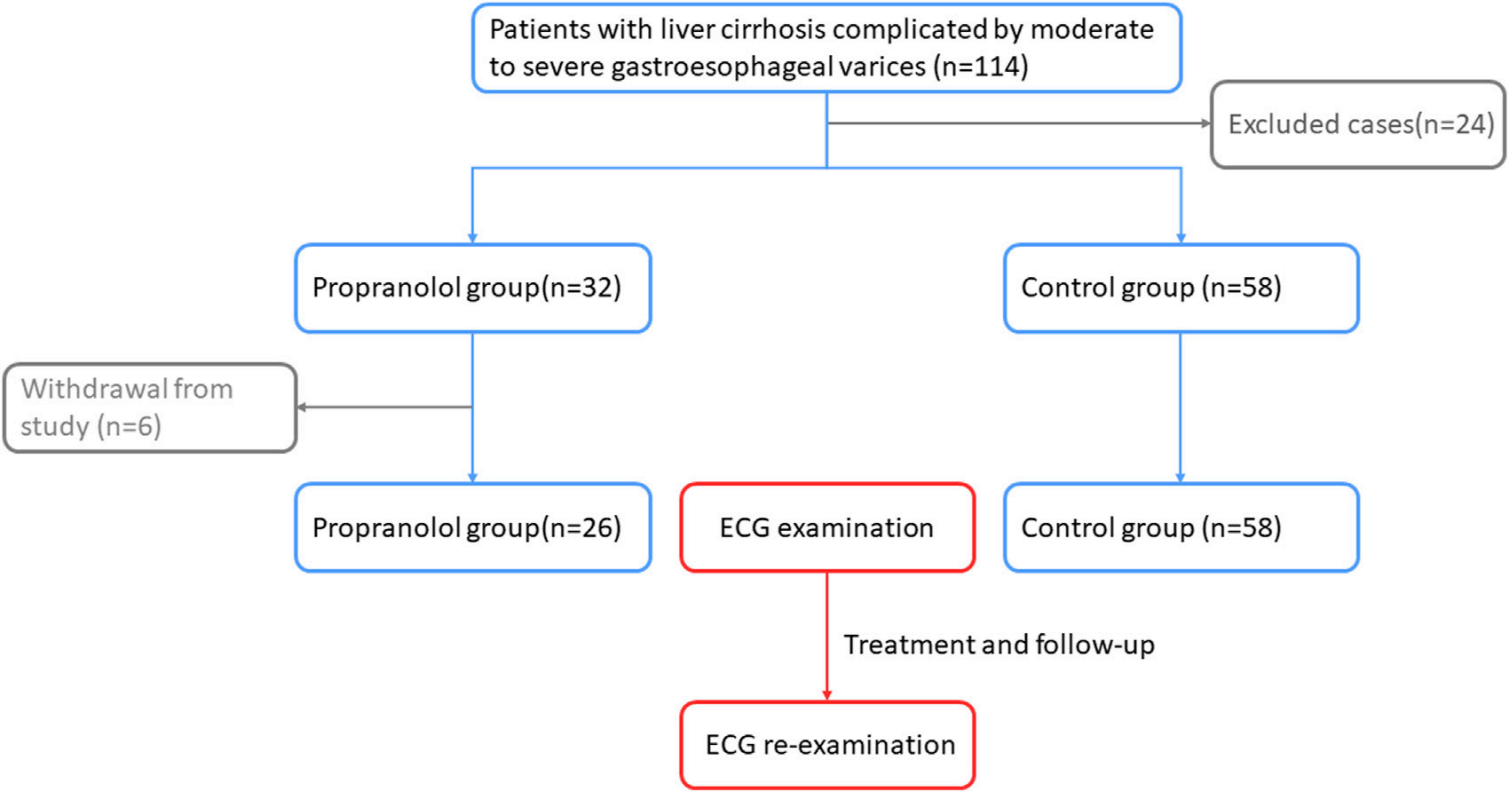
Flowchart of the subject inclusion process.

A total of 114 patients with cirrhosis complicated by moderate-to-severe gastroesophageal varices were enrolled for the study; of these, 24 patients were excluded, including two with concomitant bronchial asthma, three with severe pulmonary infections, three with sinus bradycardia, two with second-degree atrioventricular blocks, three with coronary heart diseases, two with atrial fibrillation, and nine with hepatocellular carcinomas. A total of 90 patients were eventually included in the study, with 32 patients in the propranolol group and 58 patients in the control group. During treatment, six patients in the propranolol group were excluded due to medication non-compliance. Ultimately, 26 patients in the propranolol group and 58 patients in the control group completed the study. Figure 1 shows the flowchart of the subject inclusion process.

### 3.2 Baseline status of the patients

All included patients had cirrhosis complicated by moderate-to-severe gastroesophageal varices. The baseline values of the general characteristics were compared between the propranolol and control groups; differences in the age, sex, presence or absence of alcoholism, ascites, serum total bilirubin, serum albumin, prothrombin time, serum potassium concentration, serum calcium concentration, Child–Pugh score for liver function, and QTc interval were not statistically significant (all *p*-values > 0.05). The two groups were consistent with respect to sex, age, baseline QTc interval, and other factors that could possibly affect QTc interval, and were thus comparable. Table 1 shows the baseline data of the two groups.

**TABLE 1:** Baseline data on patients with cirrhosis complicated by moderate-to-severe gastroesophageal varices.

Indicator	Propranolol group (*n* = 26)	Control group (*n* = 58)	***t*/** *χ* ^***2***^ **/*Z***	*P*
**Age (years)**	56.33 ± 10.48	56.17 ± 12.45	0.453	0.650
**Male (%)**	23 (88.5)	48 (82.823)	0.446	0.504
**Alcoholic (%)**	5 (19.2)	6 (10.3)	1.246	0.264
**Ascites (%)**	16 (61.5)	38 (65.5)	0.124	0.725
**Total bilirubin in serum (IQR, µmol/L)**	27.70 (14.78–73.28)	39.55 (24.25–92.10)	1.747	0.081
**Serum albumin (g/L)**	30.45 ± 6.84	31.06 ± 5.52	0.433	0.666
**Prothrombin time (s)**	17.05 ± 2.87	18.60 ± 4.43	1.635	0.106
**Child–Pugh score for liver function**	8.23 ± 2.67	8.79 ± 2.25	0.997	0.322
**Serum potassium concentration (mmol/L)**	3.68 ± 0.56	3.68 ± 0.72	0.036	0.972
**Serum calcium concentration (mmol/L)**	2.02 ± 0.15	2.07 ± 0.17	1.154	0.252
**QTc interval (ms)**	450.23 ± 37.18	453.74 ± 30.03	0.174	0.647

### 3.3 Changes in the QTc intervals of the propranolol group before and after treatment

**FIGURE 2:**
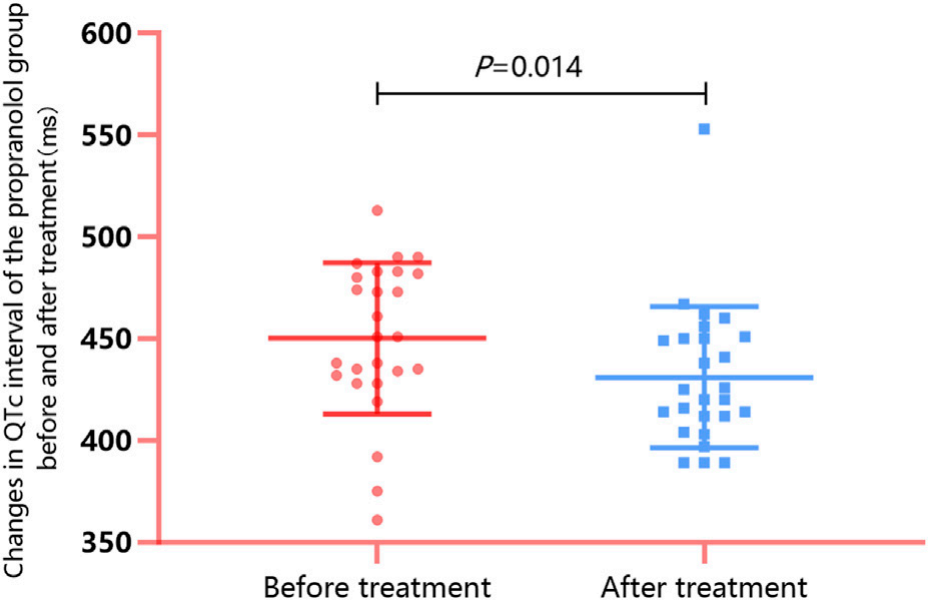
Changes in the QTc interval of the propranolol group before and after propranolol treatment: Follow-up was continued for a median duration of 7.00 days after administration of propranolol and conventional treatment. Electrocardiography reexamination revealed a decrease in the QTc interval from 450.23 ± 37.18 ms to 431.04 ± 34.64 ms (*p* = 0.014), and the number of patients with QTc interval prolongation decreased from 14 to 5 (19.2%; *p* < 0.001). Normalization of the QTc interval occurred in nine patients with QTc interval prolongation, leading to a normalization rate of 64.3% (9/14).

All 26 patients in the propranolol group underwent ECG examinations before commencing propranolol treatment. The results indicated an average baseline QTc interval of 450.23 ± 37.18 ms, among which 14 patients (53.8%) demonstrated QTc interval prolongation. The patients were treated with propranolol as well as multimodal therapy and subsequently followed up for a median duration of 7.00 days. The results of ECG reexamination after follow-up showed that the posttreatment average QTc interval was 431.04 ± 34.64 ms, which was significantly lower than the baseline value (*t* = 2.648, *p* = 0.014; Figure 2). The number of patients with QTc interval prolongation decreased to five (19.2%), and the difference compared with the baseline value was statistically significant (*χ* ^*2*^ = 6.718, *p* < 0.001). In patients with QTc interval prolongation, the QTc interval normalization rate after treatment with propranolol and multimodal therapy was 64.3% (9/14).

### 3.4 Changes in the QTc intervals of the control group before and after treatment

**FIGURE 3:**
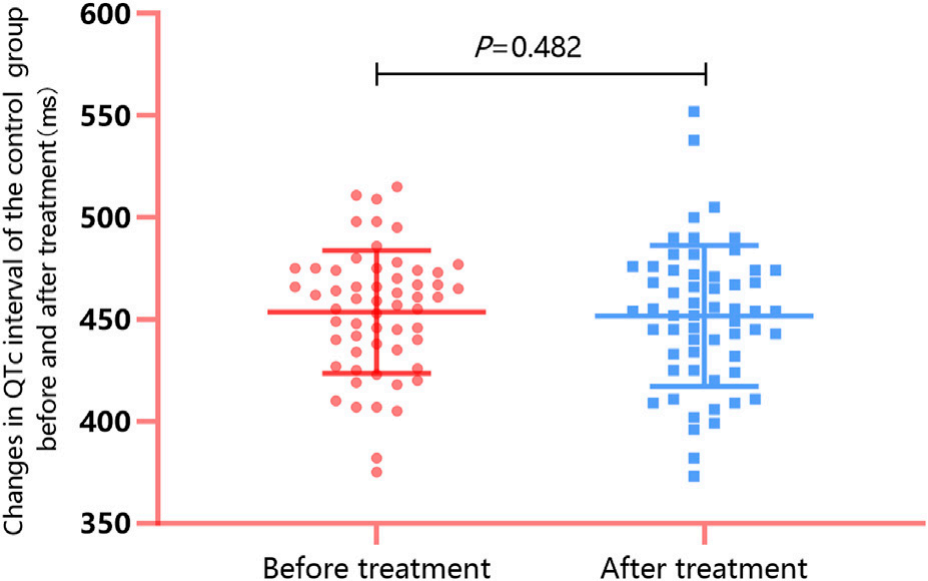
Changes in the QTc interval of the control group before and after treatment: After follow-up for a median duration of 7.50 days, the QTc interval changed from 453.74 ± 30.03 ms to 451.79 ± 34.56 ms (*p* = 0.482), and the number of patients with QTc interval prolongation decreased from 57 to 30 (51.7%; *p* = 0.457). The QTc interval normalization rate of patients with QTc interval prolongation was 10.0% (3/33).

All 58 patients in the control group underwent ECG examinations upon admission. The results indicated an average baseline QTc interval of 453.74 ± 30.03 ms, among which 33 patients (56.9%) exhibited QTc interval prolongation. The patients did not receive propranolol but received multimodal therapy based on their individual conditions; all patients were subsequently followed up for a median duration of 7.50 days. Results of ECG reexamination after follow-up demonstrated that the average posttreatment QTc interval was 451.79 ± 34.56 ms, which was not significantly different from the baseline value (*t* = 0.708, *p* = 0.482; Figure 3). The number of patients with QTc interval prolongation decreased to 30 (51.7%), and the difference compared with the baseline value was not statistically significant (*χ* ^*2*^ = 0.554, *p* = 0.457). In patients in the control group with QTc interval prolongation, the QTc interval normalization rate after treatment with propranolol-free multimodal therapy was 10.0% (3/33), which was significantly lower than that for the propranolol group (*χ* ^*2*^ = 15.75, *p* < 0.001).

### 3.5 Differences in pre- and post-treatment values of factors affecting QTc intervals in the propranolol group

To ascertain whether posttreatment QTc interval normalization in the propranolol group was primarily caused by propranolol or the improvement in liver function and correction of electrolyte imbalance after multimodal therapy, the indicator changes of factors that could affect the QTc intervals in the propranolol group before and after treatment were compared. These included items like the Child–Pugh score for liver function, serum potassium, and serum calcium. The results revealed an absence of statistically significant differences in these factors (all *p*-values > 0.05; Table 2).

**TABLE 2:** Comparison of pertinent pretreatment and posttreatment indicators in the propranolol group.

Indicator	Before treatment (*n* = 26)	After treatment (*n* = 26)	***t*/** *χ* ^***2***^ **/*Z***	*P*
**Child–Pugh score for liver function**	8.23 ± 2.67	8.08 ± 2.17	0.570	0.574
**Hepatic encephalopathy**	1 (3.8%)	0 (0)	-	1.000
**Ascites**	16 (61.5%)	15 (57.7%)	0.080	0.777
**Total bilirubin in serum (IQR, µmol/L)**	27.70 (14.78–73.28)	28.65 (13.98–77.53)	0.525	0.600
**Serum albumin (g/L)**	30.45 ± 6.84	31.24 ± 5.04	0.528	0.602
**Prothrombin time (s)**	17.05 ± 2.87	17.97 ± 3.24	1.967	0.060
**Serum potassium (mmol/L)**	3.68 ± 0.56	3.96 ± 0.59	1.773	0.088
**Serum calcium (mmol/L)**	2.03 ± 0.15	2.08 ± 0.07	1.799	0.084

### 3.6 Safety of propranolol

**FIGURE 4:**
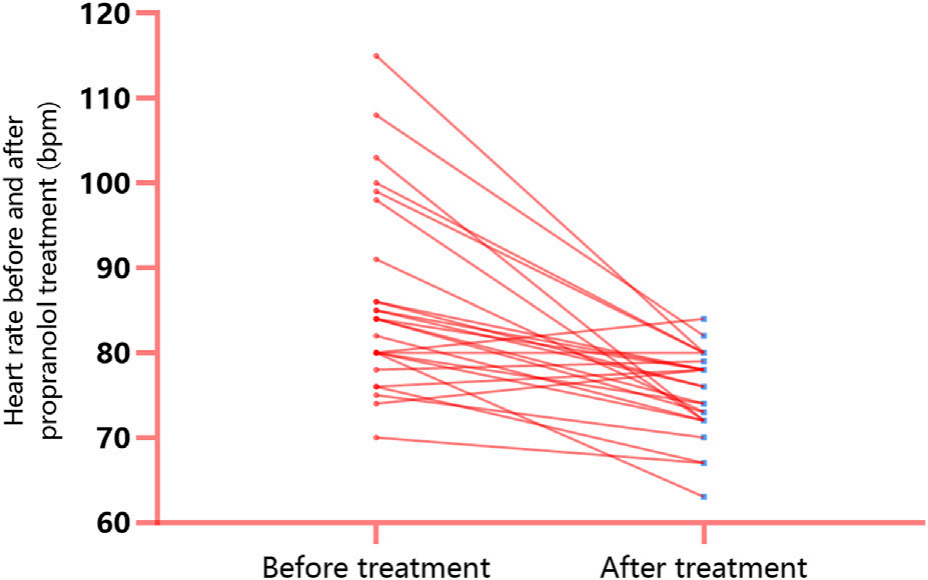
Changes in the heart rate before and after propranolol treatment: the average heart rate decreased from 86.12 ± 11.21 BPM to 75.23 ± 5.04 BPM after 1 week of propranolol treatment, and this difference was statistically significant (*t* = 5.307, *p* < 0.001). All 26 patients had heart rates >60 BPM after treatment.

Commonly reported adverse reactions to propranolol include bradycardia and bronchospasms. To assess the cardiovascular and pulmonary safety of propranolol, the changes in heart rates were monitored along with observation of the occurrence of bronchospastic attacks before and after the use of propranolol in the 26 patients in the propranolol group. The results revealed that the average heart rate decreased from 86.12 ± 11.21 beats per minute (BPM) to 75.23 ± 5.04 BPM after 1 week of propranolol treatment and that this difference was statistically significant (*t* = 5.307, *p* < 0.001; Figure 4). All 26 patients had heart rates exceeding 60 BPM after treatment, and none of the patients exhibited bronchospastic symptoms (asthma attacks, dyspnea, or bilateral wheezing).

## 4 Discussion

In the present study, incidence of QTc interval prolongation was found to be 53.8%–56.9% among patients with cirrhosis complicated by moderate-to-severe gastroesophageal varices, which is higher than that reported by Zhao et al. (2016). It is therefore evident that QT interval prolongation is the most prominent ECG characteristic of these patients, although the pathophysiological mechanisms of CCM remain unclear. Given the current lack of indicators for definite diagnosis of CCM or adequate assessment of cardiac function in CCM, QTc interval prolongation can be currently considered the one of the electrophysiological markers of CCM, although it is not the main criterion (Bernardi et al., 2012). For patients with cirrhosis and QTc interval prolongation, shortening and normalization of the QTc interval may be the key outcomes of CCM treatment and may help reduce the incidence of malignant arrhythmias and mortality. Several studies (Kazankov et al., 2019) have indicated that QT interval correction for heart rate is not associated with mortality in patients with cirrhosis; however, most of these studies support that QTc interval prolongation is associated with poor prognosis in patients with cirrhosis. Therefore, correcting long QTc intervals may have potential benefits for such patients.

There are currently no specific drug recommendations for treating QTc interval prolongation in cirrhosis. In most cases, there are attempts to correct QTc interval prolongation by correcting the associated electrolyte imbalances and improving liver function; however, clear effects have not yet been achieved. The results of this study reveal that propranolol-free conventional treatment in the short term is unable to correct QTc interval prolongation; therefore, there is considerable potential for exploring pharmacological treatment of QTc interval prolongation in cirrhosis.

Extant literature studies indicate that alcohol consumption, liver function, serum calcium, and serum potassium may be some of the important factors influencing the QTc interval. Therefore, the factors that may affect QTc intervals were compared between the propranolol and control groups in this study; it was found that the baseline characteristics of the two groups were relatively consistent, particularly with regard to the incidence of QTc interval prolongation.

The results of this work indicate that under the premise that the conventional treatment for cirrhosis was administered to both groups of patients, short-term treatment using propranolol resulted in a decrease in the average QTc interval and significant lowering of the incidence of QTc interval prolongation (53.8%–19.2%) in patients with cirrhosis, which is an important finding. Comparisons were also performed for the liver function and electrolyte levels in the propranolol group before and after treatment, for which no significant differences were observed in the Child–Pugh score for liver function, serum potassium, and serum calcium. This again suggests that the shortening of the QTc interval and significant reduction in the incidence of QT interval prolongation in the propranolol group are not caused by the multimodal therapy, improvement in liver function, or correction of electrolyte imbalances but are attributable to the effects of propranolol treatment.

The commonly expected adverse reactions to propranolol include bradycardia and bronchospasms. The patients in the propranolol group were monitored for potential adverse reactions after short-term propranolol treatment, and they did not show any obvious bradycardia or bronchospastic diseases. However, the safety of using non-selective β-blockers in patients with cirrhosis in the decompensation stage remains debatable. Previous studies (Alvarado-Tapias et al., 2020; Tellez et al., 2020) have reported that the use of propranolol in patients with decompensated cirrhosis or cirrhosis complicated by refractory ascites may lead to adverse effects, such as decreased cardiac output and kidney injury. In contrast, a meta-analysis by Senzolo et al. (2009) demonstrated that propranolol treatment was safe and had no effects on hemodynamics in patients with cirrhosis with spontaneous bacterial peritonitis. Nonetheless, the safety of propranolol in patients with cirrhosis, including those with QT interval prolongation, requires further continuous assessment in clinical practice. In this study, propranolol was only used in the short term, and an earlier pharmacological study (Arthur et al., 1985) has shown that the drug concentration of orally administered propranolol in patients with cirrhosis could reach a “steady state” after 72 h. Therefore, the authors believe that a median course of 7 days is sufficient to reflect the therapeutic effects; however, the question of whether long-term treatment produces different effects needs further study.

Regardless of the findings, this study has some limitations. The impact of carvedilol on QTc interval was not included in the study, as propranolol is a more accessible drug. Furthermore, the number of cases examined in this study is relatively small, and subgroups with different severe cirrhosis were not distinguished.

In conclusion, the short-term use of propranolol aids in the correction of QT interval prolongation and provides positive therapeutic value for CCM in patients with cirrhosis complicated by moderate-to-severe gastroesophageal varices and QT interval prolongation. However, the safety of propranolol in these patients, especially those with severely decompensated cirrhosis, must still be evaluated and observed meticulously. In patients with cirrhosis with QT interval prolongation but without gastroesophageal varices, the use of propranolol for treatment requires comprehensive evaluation as well as further research for confirmation.

## Data Availability

The original contributions presented in the study are included in the article/Supplementary material; further inquiries can be directed to the corresponding authors.

## Abbreviations

BPM, beats per minute; CCM, cirrhotic cardiomyopathy; ECG, electrocardiogram; QTc, corrected QT.
